# Inhibition of discoidin domain receptor (DDR)-1 with nilotinib alters CSF miRNAs and is associated with reduced inflammation and vascular fibrosis in Alzheimer’s disease

**DOI:** 10.1186/s12974-023-02802-0

**Published:** 2023-05-16

**Authors:** Max Stevenson, Rency Varghese, Michaeline L. Hebron, Xiaoguang Liu, Nick Ratliff, Amelia Smith, R. Scott Turner, Charbel Moussa

**Affiliations:** 1 Translational Neurotherapeutics Program, Laboratory for Dementia and Parkinsonism, Department of Neurology, Georgetown University Medical Center, Building D, Room 265, 4000 Reservoir Rd, NW, Washington, DC 20057 USA; 2 Genomics and Epigenomics Shared Resource, Department of Oncology, Georgetown University Medical Center, Building D, 4000 Reservoir Rd, NW, Washington, DC 20057 USA; 3 Memory Disorders Program, Department of Neurology, Georgetown University Medical Center, 4000 Reservoir Rd, NW, Washington, DC 20057 USA

**Keywords:** Discoidin domain receptor 1, Alzheimer’s disease, MicroRNAs, Collagen, Nilotinib, Cytokines, Chemokines

## Abstract

Discoidin Domain Receptor (DDR)-1 is activated by collagen. Nilotinib is a tyrosine kinase inhibitor that is FDA-approved for leukemia and potently inhibits DDR-1. Individuals diagnosed with mild–moderate Alzheimer’s disease (AD) treated with nilotinib (versus placebo) for 12 months showed reduction of amyloid plaque and cerebrospinal fluid (CSF) amyloid, and attenuation of hippocampal volume loss. However, the mechanisms are unclear. Here, we explored unbiased next generation whole genome miRNA sequencing from AD patients CSF and miRNAs were matched with their corresponding mRNAs using gene ontology. Changes in CSF miRNAs were confirmed via measurement of CSF DDR1 activity and plasma levels of AD biomarkers. Approximately 1050 miRNAs are detected in the CSF but only 17 miRNAs are specifically altered between baseline and 12-month treatment with nilotinib versus placebo. Treatment with nilotinib significantly reduces collagen and DDR1 gene expression (upregulated in AD brain), in association with inhibition of CSF DDR1. Pro-inflammatory cytokines, including interleukins and chemokines are reduced along with caspase-3 gene expression. Specific genes that indicate vascular fibrosis, e.g., collagen, Transforming Growth Factors (TGFs) and Tissue Inhibitors of Metalloproteases (TIMPs) are altered by DDR1 inhibition with nilotinib. Specific changes in vesicular transport, including the neurotransmitters dopamine and acetylcholine, and autophagy genes, including ATGs, indicate facilitation of autophagic flux and cellular trafficking. Inhibition of DDR1 with nilotinib may be a safe and effective adjunct treatment strategy involving an oral drug that enters the CNS and adequately engages its target. DDR1 inhibition with nilotinib exhibits multi-modal effects not only on amyloid and tau clearance but also on anti-inflammatory markers that may reduce cerebrovascular fibrosis.

**Supplementary Information:**

The online version contains supplementary material available at 10.1186/s12974-023-02802-0.

## Background

Vascular contributions to cognitive impairment are increasingly recognized [1–6]. Small vessel disease of the brain may contribute to all dementias, including Alzheimer’s disease (AD) [5–7]. Vascular changes in AD have been attributed to the vasculo-toxic effects of β-amyloid (Aβ) [8]. Early cognitive dysfunction is associated with hippocampal capillary damage and blood–brain barrier (BBB) breakdown irrespective of Aβ and/or tau pathologies [9]. Animal studies suggest that Aβ and tau lead to blood vessel abnormalities and BBB breakdown [8, 10–12]. However, the development of neurovascular dysfunction and its relationship with Aβ and tau in the dementia continuum are not well understood.

Discoidin Domain Receptor (DDR)-1 is a non-receptor tyrosine kinase (TK) that is implicated in several fibrotic disorders. DDR1 inhibition reduces accumulation of macrophages, attenuates the inflammatory response and suppresses fibrosis in models of atherosclerosis [13, 14], and renal and lung fibrosis [15, 16]. DDR1 is expressed in the CNS and its activation increases microglial activity and matrix metalloproteases (MMPs), leading to BBB breakdown [17]. DDR1 inhibition stimulates clearance of neurotoxic proteins via autophagy in animal models of neurodegeneration [18–22] and DDR1 is upregulated in post-mortem Parkinson’s disease (PD) and AD brains [18, 19, 23–29]. Knockdown of DDR1 via shRNA reduces Aβ levels and prevents cell loss in vivo and in vitro [25]. Whole genome miRNA sequencing of CSF from patients diagnosed with PD with mild cognitive impairment (PD–MCI) and treated with the DDR1 inhibitor, nilotinib, demonstrates changes of molecular pathways that regulate angiogenesis and the BBB, neuro-inflammation, and protein clearance via autophagy [30–32]. Changes of miRNAs that control angiogenesis and the BBB are significantly correlated with motor and cognitive stability in PD–MCI patients [31].

To address the question of DDR1 effects on blood vessels and inflammation in dementia, we performed longitudinal epigenomic studies via unbiased next generation whole miRNA genome sequencing of CSF obtained from mild–moderate AD patients who were treated with nilotinib (versus placebo), which significantly reduced CSF Aβ levels and CNS amyloid burden as measured with Positron Emission Tomography (PET) [33].

## Materials and methods

### Study design

The primary research objective of this study was to assess longitudinal alterations in CSF microRNAs from samples previously collected in mild–moderate AD patients [33]. As previously described, AD patients [33] were randomized to placebo for 12 months (*n* = 20) or nilotinib, 150 mg for 6 months followed by nilotinib, 300 mg, for another 6 months (*n* = 17). All enrollees (*N* = 37) met inclusion/exclusion criteria and the study excluded cardiovascular disease, mini mental status exams (MMSE) score out of range, or a diagnosis of AD not supported by biomarker evidence. Concomitant drugs known to prolong the QTc interval and history of any cardiovascular disease, including myocardial infraction or cardiac failure, angina, arrhythmia were not permitted. Any cardiovascular or cerebrovascular event (e.g., myocardial infarction, unstable angina, or stroke), congestive heart failure, second- or third-degree atrioventricular block, sick sinus syndrome, or other serious cardiac rhythm disturbances were excluded. Significant pathological findings on brain MRI (historical in the last 12 months prior to enrollment), including cerebral microhemorrhages, cerebral macro-hemorrhages, or superficial siderosis with vasogenic edema were excluded. Other excluded MRI findings included more than 4 micro hemorrhages (defined as 10 mm or less at the greatest diameter); a single macro hemorrhage greater than 10 mm at greatest diameter; evidence of cerebral contusion, encephalomalacia, aneurysms, vascular malformations, or infective lesions; evidence of multiple lacunar infarcts or stroke involving a major vascular territory, severe small vessel, or white matter disease; space occupying lesions; or brain tumors. There was two diabetic patients in this study. Mean (SD) age of the participants was 70.7 years (6.48) and the sample included 10 men (27%) and 27 women (73%). The mean (SD) MMSE was 19.2 (3.1) in the placebo group and 19.8 (2.5) in the nilotinib group. The apolipoprotein-E (APOE) genotype in the placebo group was E4/E4 5 (25%), E3/E4 5 (25%), E2/E4, 4 (20%), E2/E2 1 (5%), and 5 (25%) inconclusive genotypes. In the nilotinib group, APOE was E4/E4 7 (41.2%), E3/E4 1 (5.9%), E2/E4 3 (17.6%), E3/E3 1 (5.9%), E2/E2 1 (5.9%), and 4 (23.5%) inconclusive genotypes. Participants and study partners were recruited, and this study was conducted in accordance with Good Clinical Practice guidelines and approved by the Institutional Review Board (IRB #2016–0351) at GUMC as well as GHUCCTS scientific review board. The study was conducted under FDA Investigational New Drug (IND) #130732 and registered in ClinicalTrials.gov (NCT02947893) [33]. All samples collected and used for experiments presented in this study were de-identified and relabeled with a study ID.

### Cerebrospinal fluid microRNA sequencing and analysis

CSF was collected via lumbar puncture (LPs), which was performed on all patients between 1 and 4 h at baseline and 12 months (end of treatment) with placebo or nilotinib. Cell-free total RNA was isolated from 200 µl of CSF using the Qiagen miRNAeasy extraction kit (Qiagen, 217,184) and CSF microRNA sequencing was performed as we previously explained [32]. Samples were normalized to an input volume of 5 μl RNA eluate to prepare miRNAseq libraries using Qiagen QiaSeq miRNA-seq library preparation kit (Qiagen, 331,502). Next-generation sequencing (NGS) was performed on a NextSeq 550 Sequencing System (Illumina) using single-end (SE) 1 × 75 base pairs (bp) sequencing to a depth of 25 million raw reads per sample.

### Statistical analysis

Data analysis was performed using Partek Flow software to identify differentially expressed microRNAs. An optimized workflow was used to perform quality assessment, alignment, quantification, normalization, and different analysis. Quality assessment of data in fastq format was performed in Partek flow and reads containing the adapters or low-quality bases (Qscore ≤5) were removed. The trimmed reads were then aligned to the hg38 reference genome using the Bowtie method. The aligned reads were then quantified to the miRBase reference to obtain the total miRNA read count for each sample. DESeq2 method was used for normalization of the miRNA read counts, followed by DESeq2 implementation of the likelihood ratio test (LRT) to test for multiple terms at once and interaction between the variables. The LRT for count data is conceptually similar to an analysis of variance (ANOVA) calculation, except that in the case of the Negative Binomial GLM, an analysis of deviance (ANODEV) is used, where the deviance captures the difference in likelihood between a full and a reduced model [34]. The *p* values obtained are then adjusted for multiple testing using the Benjamini and Hochberg method. Gene ontology (GO) pathway and functional enrichment analyses were performed on the gene targets of the significant DEMs using PANTHER Classification System. Only enriched pathways and GO terms that met Benjamini–Hochberg *p* < 0.05 were reported.

### Measurement of CSF DDR1 activity

To estimate spectrophotometrically the levels of active or phosphorylated DDR1 (pDDR1) in CSF, we measured the level of pan-tyrosine pDDR1 via ELSA using PathScan phosphorylated DDR1 panTyrosine (Cell Signaling, KIT# 7863) and determined the absorbance (AU) change during the assay. To perform longitudinal measurement of CSF pDDR1 over time within the same patient, absorbance measurement of CSF pDDR1 collected at baseline was subtracted from absorbance (AU) at the next CSF collection and estimated the change of linear absorbance versus time of assay mixture measured at wavelength 450 nm. We plotted the linear graph with absorbance change versus time that is the absorbance change per time of the reaction (ΔA/time) and found the slope (*m*) that is *y* = mx + *c* using linear regression analysis as a convenient mathematical expression. Slower slope suggests less active DDR1 (less pDDR1), and faster slope is more active DDR1 (more pDDR1) over time.

### Plasma biomarkers

Plasma Aβ40, Aβ42, total tau, and p-tau181 and p-tau217 were measured using Biofluid Biomarker Assay via SQUID IMR (Immuno Magnetic Reagents) Platform (MagQu Co., Ltd) that uses UMI magnetic reagents (Cat # AB0-0068, MagQu) and Aβ40, Aβ42, total tau, and p-tau 181 and p-tau217 IMR reagent (Cat. #MF-ASC-006B; MagQu).

### Data availability

The data supporting the current study are available from the corresponding author on request.

## Results

### miRNAs that target DDR1 are significantly altered by nilotinib treatment.

**Fig. 1:**
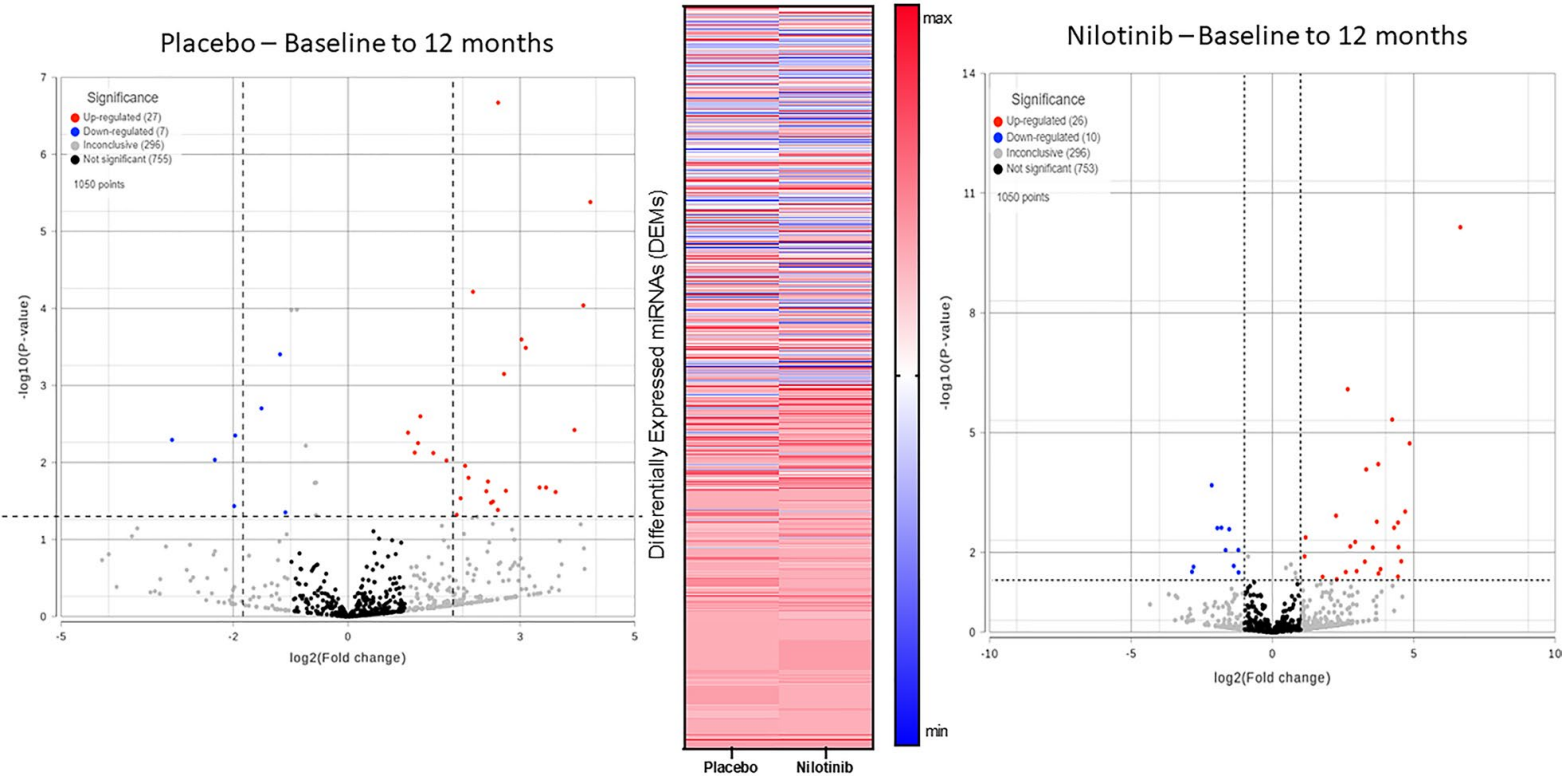
Unbiased next generation whole genome sequencing of CSF miRNA showed expression profiles of a total of 1050 miRNA between baseline and 12 months in the placebo (n = 11) and nilotinib groups (n = 12) (two-way ANOVA)

Unbiased next generation whole genome sequencing of CSF miRNA showed expression profiles of a total of 1050 miRNA (Fig. 1) between baseline and 12 months in the placebo (*n* = 11) and nilotinib groups (*n* = 12). A total of 17 microRNAs (1.6%) were significantly different between the placebo and nilotinib groups, accounting for both treatment and time. Ingenuity Pathway Analysis (IPA) microRNA Target filter tool matched associated mRNAs or targets that are experimentally validated from TarBase, miRecords, as well as highly predicted miRNA–mRNA interactions from TargetScan and revealed that these microRNAs are associated with genes that primarily control molecular pathways of autophagy, inflammation, collagen expression, and dopamine metabolism.

### DDR1 is longitudinally inhibited in the CSF of AD patients treated with nilotinib

**Fig. 2:**
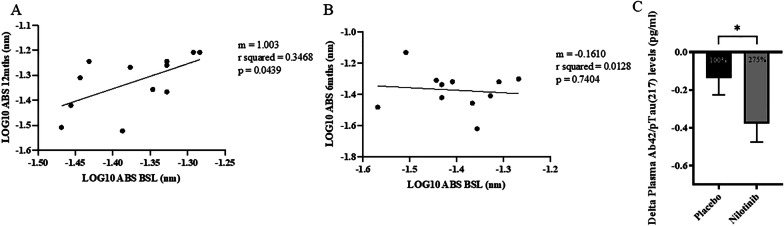
DDR1 is longitudinally inhibited in the CSF of AD patients treated with nilotinib. ELISA measurement of pan-tyrosine phosphorylated DDR1 (pDDR1) shows the slope (m) of log_10_ linear regression of mean difference values in the CSF of **A**) AD patients from baseline-end of treatment, indicating (rising slopes) an increase in active pDDR1 level over time. Nilotinib treatment **B**) results in slower slope (m) of log_10_ linear regression of mean difference values between baseline and end of treatment in AD patients treated with nilotinib, indicating reduction of pDDR1 (active). N = 12 placebo and n = 12 in nilotinib. **C**) measurement of plasma Aβ42 and pTau217 using Biofluid Biomarker Assay via SQUID IMR (Immuno Magnetic Reagents) Platform (MagQu Co., Ltd). N = 12 placebo and N = 10 nilotinib. Graphed as mean ± standard error of the mean. The changes in the ratio of Aβ42/pTau (217) across each group were compared using a one‐tailed unpaired t‐test with Welch's corrections. Asterisks denote actual P‐value significances (*P < 0.05) between groups and are noted in the figure. Statistical analysis was performed using GraphPad Prism, version 9.1.2 (GraphPad Software Inc.)

**Fig. 3:**
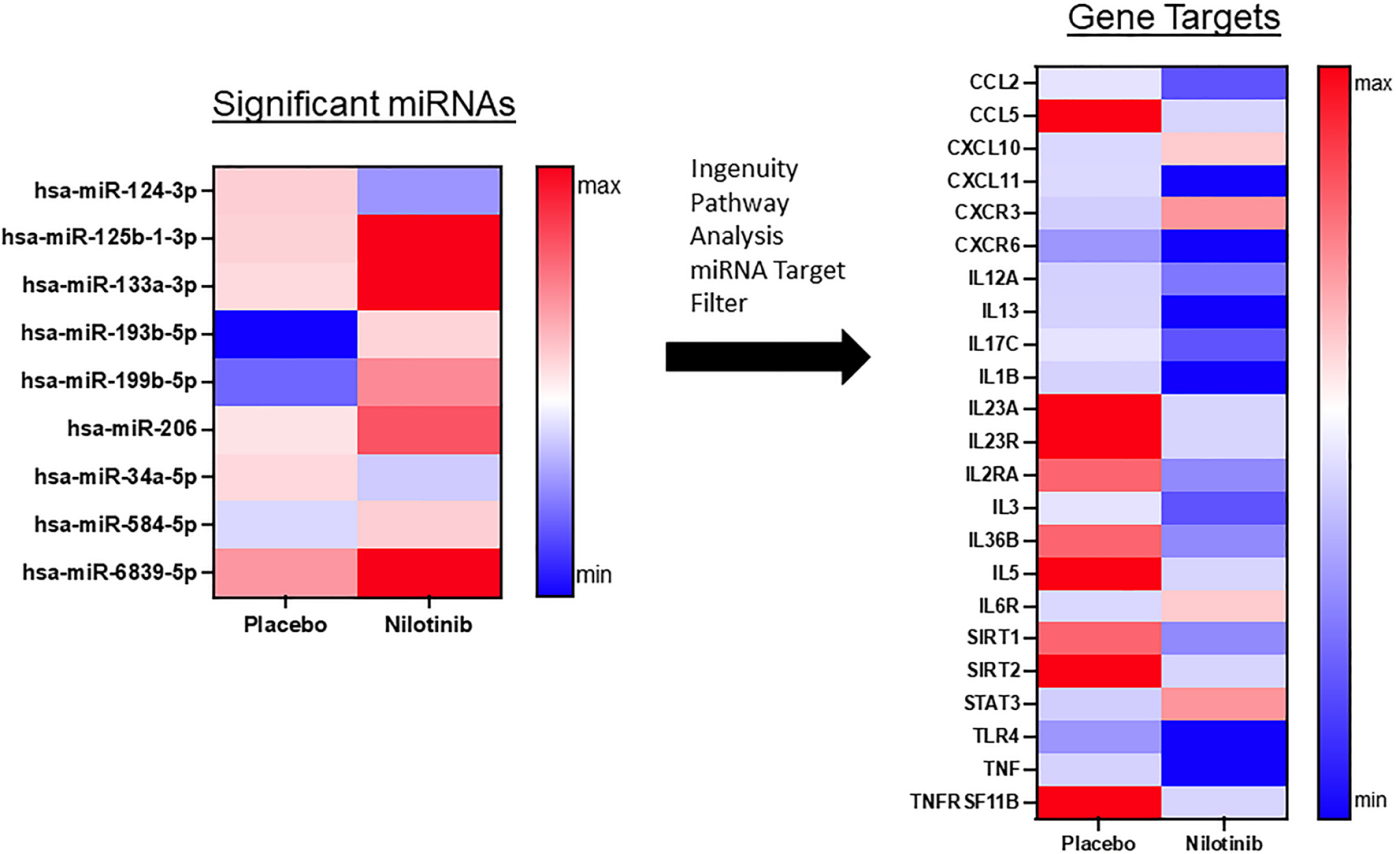
miRNAs that target inflammation are significantly reduced in nilotinib treated patients. A total of 17 microRNAs (1.6%) were significantly different between the placebo and nilotinib groups, accounting for both treatment and time. Ingenuity Pathway Analysis of microRNA matched associated mRNAs or targets that are experimentally validated from TarBase, miRecords, as well as highly predicted miRNA–mRNA interactions from TargetScan and revealed that 9 of these microRNAs are associated with genes that regulate inflammation. N = 11 placebo, n = 12 nilotinib

To verify the effects of nilotinib on DDR1 activity, we show that the level of CSF pDDR1 in placebo-treated AD patients (Fig. 2A) was significantly increased between baseline and end of treatment (slope = 1.003, *p* = 0.04). However, treatment of AD patients with the DDR1 inhibitor nilotinib (Fig. 2B) showed a remarkably slower (> 10x) and negative slope (slope = -0.1610, *p* = 0.7). We previously demonstrated that DDR1 inhibition with nilotinib lowers CSF Aβ levels and reduces CNS amyloid burden by PET in the same cohort of AD patients [33]. Here, we show that the ratio of plasma Aβ42/ptau217 is also significantly reduced with nilotinib compared to placebo (Fig. 3C), suggesting alterations of amyloid and tau levels, in agreement with previous findings [33]. A specific miRNA (hsa-miR-199b-5p) that targets DDR1 gene expression is reduced in the placebo versus nilotinib groups (Fig. 3), showing downregulation of DDR1 expression in the nilotinib versus placebo.

### miRNAs that target inflammation are reduced in nilotinib treated patients

We observed changes in specific miRNAs that control interleukins (ILs) and chemokines that are collectively indicative of reduced inflammation with nilotinib treatment (Fig. 3). Specific miRNAs that target genes related to pro-inflammatory cytokines were enhanced following nilotinib treatment (Fig. 3), including but not limited to IL1β (hsa-miR-125b-1-3p), IL2 (hsa-miR-199b-5p), IL3 (hsa-miR-206), IL5 (hsa-miR-193b-5p), IL12 (hsa-miR-96-5p), IL13 (hsa-miR-125b-1-3p), IL17 (hsa-miR-206), IL23 (hsa-miR-193b-5p, hsa-miR-199b-5p), IL36 (hsa-miR-199b-5p), resulting in decreased expression, while the anti-inflammatory IL6 (hsa-miR-34a-5p) was increased. IL-6 and signal transducer and activator of transcription 3 (STAT3) (hsa-miR-124-3p), a signaling pathway in the regulation of macrophage polarization, M1-type and M2-types, are both upregulated with nilotinib, suggesting an overall change in the anti-inflammatory profile. Expression of the pro-inflammatory tissue necrosis factor (TNF) (hsa-miR-125b-1-3p) and its receptors (TNFRs) (hsa-miR-193b-5p), in addition to toll-like receptor (TLR-4) (hsa-miR-6839-5p) are also reduced in nilotinib-treated patients. There is significant regulation of CCL2 (hsa-miR-206), CCL5 (hsa-miR-193b-5p, hsa-miR-584-5p), and CXCL10 (hsa-miR-34a-5p), CXCL11 (hsa-miR-133a-3p), CXCR3 (hsa-miR-124-3p), and CXCR6 (hsa-miR-6839-5p).

### miRNAs that target vascular fibrosis are reduced in nilotinib treated patients

**Fig. 4:**
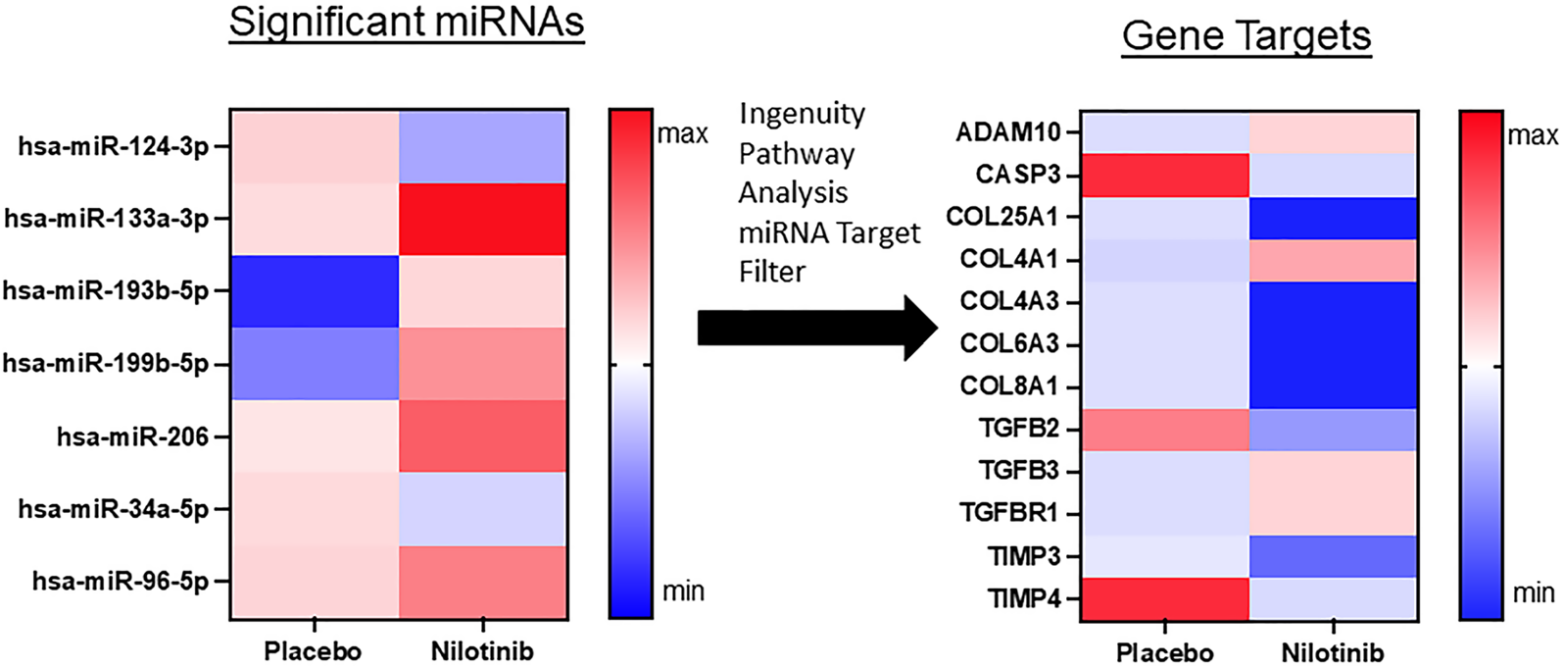
miRNAs that target vascular fibrosis are significantly reduced in nilotinib treated patients**.** A total of 17 microRNAs (1.6%) were significantly different between the placebo and nilotinib groups, accounting for both treatment and time. Ingenuity Pathway Analysis of microRNA matched associated mRNAs or targets that are experimentally validated from TarBase, miRecords, as well as highly predicted miRNA–mRNA interactions from TargetScan and revealed that 7 of these microRNAs are associated with genes that regulate collagen expression. N = 11 placebo, n = 12 nilotinib

Specific miRNAs that target collagens (hsa-miR-133a-3p, hsa-miR-96-5p) were increased in the nilotinib group (Fig. 4), indicating a decrease in expression of collagens 25, 4, 6, and 8. There are differential changes between nilotinib and placebo groups in transforming growth factor-beta 2 (TGF-β2) (hsa-miR-199b-5p) and 3 (hsa-miR-34a-5p) and an increase with nilotinib in expression of the corresponding receptor (TGFBR1) (hsa-miR-34a-5p), which is a major mediator of tissue fibrosis and regulation of MMPs, suggesting reduced effects on fibrosis. Significant increases in miRNAs (hsa-miR-206, hsa-miR-193b-5p) that target Tissue Inhibitors of Metalloproteases (TIMP)3 and TIMP4 activity that regulate amyloid precursor protein (APP) cleavage and are expressed at increased levels in AD brains, indicate a normalization of TIMP expression—further supporting the hypothesis that DDR1 inhibition promotes neurovascular health. ADAM10 (A Disintegrin and Metalloproteinase), known as α-secretase that plays a critical role in APP cleavage, is downregulated in AD but is increased (hsa-miR-34a-5p) in nilotinib-treated patients. Notably, expression of critical markers of apoptosis such as caspase-3 (hsa-miR-193b-5p) and BCL2 (hsa-miR-206) are reduced with nilotinib. To validate the effects of DDR1 on collagen expression in a mouse model of vascular angiopathy (See Additional file 1), we generated DDR1 knockout mice (TgAPP^tg/tg^/DDR^−/−^) and showed that vascular collagen IV (Additional file 2: Fig. S1) was significantly reduced along the endothelial lining of blood vessels in TgAPP^tg/tg^/DDR^−/−^, resulting in increased blood vessel diameter, compared to TgAPP^tg/tg^/DDR^+/+^ littermate controls.

### miRNAs that target DDR1 and autophagy are significantly altered by nilotinib treatment

**Fig. 5:**
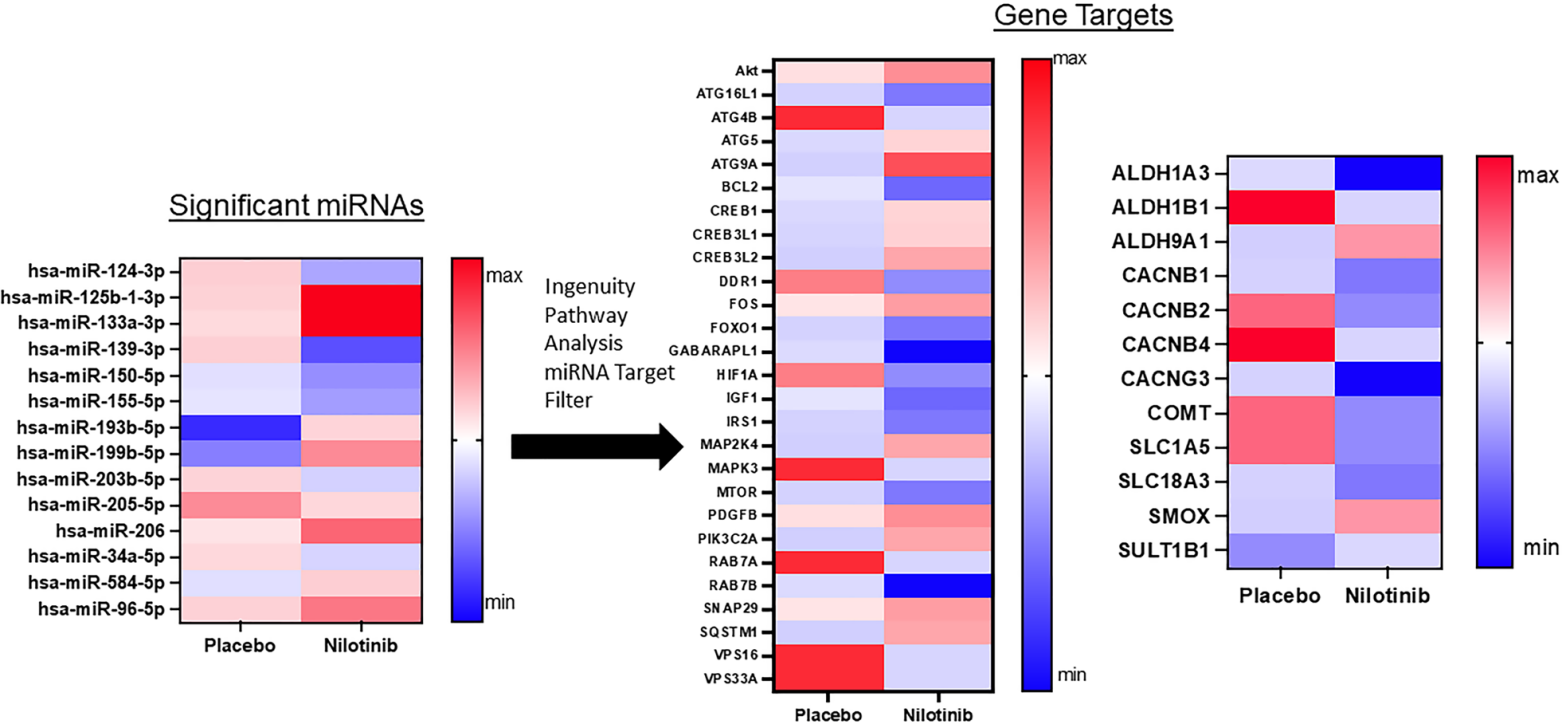
miRNAs that target autophagy and dopamine metabolism are significantly altered between placebo and nilotinib. A total of 17 microRNAs (1.6%) were significantly different between the placebo and nilotinib groups, accounting for both treatment and time. Ingenuity Pathway Analysis of microRNA matched associated mRNAs or targets that are experimentally validated from TarBase, miRecords, as well as highly predicted miRNA–mRNA interactions from TargetScan and revealed that 14 of these microRNAs are associated with genes that control autophagy and dopamine metabolism. *N* = 11 placebo, *n* = 12 nilotinib

In addition, specific miRNAs that target autophagy-associated genes (ATGs) were altered to promote autophagy flux in the nilotinib group when compared to the placebo group (Fig. 5). These miRNAs and ATG changes include downregulation of ATG16L1 (hsa-miR-96-5p) that inhibits autophagosome formation, and ATG4B (hsa-miR-193b-5p), that downregulates autophagy [35]. Upregulation of AKT (hsa-miR-124-3p, hsa-miR-150-5p) is known to enhance ATG4B activity, which is a key regulator of the LC3/GABA-A Receptor associated protein GABARAPL1 (hsa-miR-133a-3p) that interacts with the family of ATG8 (also known as LC3)—indispensable for autophagosome formation and maturation [35]. ATG9A (hsa-miR-139-3p) is critical for autophagosome formation and ATG5 (hsa-miR-34a-5p) is an integral part of the ATG5–ATG12–ATG16L1 complex that catalyzes ATG8/LC3 lipidation for autophagosome formation, and maturation [35]. Interestingly, positive markers of autophagic flux in the nilotinib group are also associated with downregulation of BCL2 (hsa-miR-206), that binds to Beclin 1, thus preventing assembly of the pre-autophagosome structure and inhibiting autophagy and apoptosis. Furthermore, inhibition of the mammalian target of rapamycin (mTOR) (hsa-miR-96-5p), which is downregulated by nilotinib, is associated with induction of autophagy [35]. Several endosome-related genes, i.e., protein vesicle trafficking via RAB7A and RAB7B (hsa-miR-193b-5p, hsa-miR-133a-3p), the autophagosome cargo known as ubiquitin-binding protein p62 (SQSTM1) (hsa-miR-124-3p), and Vacuolar Sorting genes VPS16 and VPS33A (hsa-miR-193b-5p) and synaptosome associated protein (SNAP) 29 (hsa-miR-155-5p) are also differentially regulated between the placebo and nilotinib groups. Collectively, these changes are indicative of autophagosome maturation and autophagy flux, membrane trafficking and cellular transport [35].

We also observed upregulation of the nuclear transcription factor CREB (hsa-miR-34a-5p, hsa-miR-203b-5p), that binds to cAMP response element (CRE) to regulate neuronal processes, including metabolism and survival and expression of different transcription factors and growth factors. Platelet derived growth factor (PDGF)B (hsa-miR-150-5p) is upregulated in the nilotinib group. There is also a significant decrease in insulin growth factor (IGF), (hsa-miR-206) and Insulin Receptor Signaling (IRS1), (hsa-miR-96-5p) that control main pathways of insulin signaling, such as the phosphatidylinositol 3-kinase (PI3K) (hsa-miR-124-3p), Akt (hsa-miR-150-5p), Akt substrates, Ras and mitogen-activated protein (MAP) (hsa-miR-124-3p, hsa-miR-193b-5p) kinase. Hypoxia-Inducible Factor (HIF1A) (hsa-miR-199b-5p), and the Forkhead/winged helix box gene (FOXO) (hsa-miR-96-5p) are involved in energy metabolism, oxidative stress, proteostatis, apoptosis, immunity, and inflammation, and are downregulated, whereas, expression of the immediate early gene, Fos (hsa-miR-155-5p), that is critical to learning and memory in increased with nilotinib.

### miRNAs that target dopamine and acetylcholine metabolism are significantly altered between placebo and nilotinib

We previously demonstrated that AD patients treated with nilotinib exhibit a decrease in dopamine metabolism following 12 months of treatment with nilotinib versus placebo [33]. Here, we observe inhibition of Catechol-O-methyltransferase (COMT) (hsa-miR-199b-5p) which is one of several enzymes that degrade catecholamine, including dopamine (Fig. 5). Interestingly, SLC18A3 (hsa-miR-96-5p), the vesicular acetylcholine transporter, and VMAT1, the vesicular monoamine (dopamine) transporter (SLC1A5) (hsa-miR-199b-5p) are reduced by nilotinib, suggesting prolonged synaptic presence of these neurotransmitters prior to active transport into synaptic vesicles. There is an increase in sulfotransferases (SULT1B1) (hsa-miR-205-5p), which mediates sulfonation or detoxification of dopamine. There is an increase in spermine oxidase (SMOX) (hsa-miR-124-3p, hsa-miR-34a-5p) that is involved in polyamine catabolism. We also observed upregulation of the nuclear transcription factor (CREB) (hsa-miR-34a-5p, hsa-miR-203b-5p) that binds to cAMP response element (CRE), to regulate neuronal processes, including metabolism and survival and expression of different transcription factors and growth factors. Expression of several components of a calcium channel complex consisting of the ancillary subunits CACNB1 (hsa-miR-96-5p), CACNB2 (hsa-miR-199b-5p, hsa-miR-584-5p), CACNB4 (hsa-miR-193b-5p, hsa-miR-96-5p, and CACNG3 (hsa-miR-125b-1-3p) are decreased, suggesting reduced calcium-induced toxicity. Changes in expression of several subunits or isozymes of Aldehyde Dehydrogenase 1, including ALDH1A (hsa-miR-133a-3p), ALDH1B (hsa-miR-193b-5p), and ALDH9A (hsa-miR-124-3p), that are associated with detoxification, energy metabolism and Gamma-Amino Butyric Acid (GABA) metabolism were also observed.

## Discussion

The current study is a continuation of our previous phase 2 investigation in mild–moderate AD patients treated with the DDR1 inhibitor, nilotinib, which significantly lowered CSF Aβ levels and amyloid burden as measured by PET [33]. Nilotinib inhibits Abelson (IC_50_ > 20 nM) [36] and is FDA-approved for Philadelphia chromosome Chronic Myelogenous Leukemia (CML) that results from Abelson (Abl) mutations at oral doses of 300 mg twice daily. However, nilotinib more potently inhibits DDR1 (IC_50_ = 1 nM) [36]. Pharmacologically, nilotinib was measured in the CSF and plasma of AD patients at the 150 mg daily dosage (C_max_: 3.46 nM and 1099 nM, respectively) and the 300 mg daily dosage (C_max_: 4.7 nM and 1410 nM, respectively), suggesting a linearly proportional increase to dose in the brain [33] that would be sufficient to inhibit DDR1 (IC_50_ 1 nM). Here, we show that treatment with nilotinib significantly increases CSF miRNAs that inversely regulate DDR1 mRNA expression to decrease active CSF pDDR1, indicating a pharmacokinetic and pharmacodynamic relationship. Nilotinib penetrates the CNS (> 1 nM) and directly inhibits DDR1 and alters AD biomarkers, including Aβ as measured by PET, CSF Aβ42 and Aβ40, and the plasma ratio of Aβ42 to p-Tau217. Clinically, nilotinib (Tasigna) 150 mg and 300 mg, resulted in stabilization of cognitive and motor decline in PD–MCI over 27 months [31]. In AD, nilotinib, 150 mg may influence executive functions, but the higher 300 mg dose increases agitation and irritability according to caregiver reports [33]. Importantly, miRNA data demonstrate reduction of chemokines and cytokines and other inflammatory markers in the CSF of nilotinib-treated AD patients compared to placebo suggesting reduction of inflammation, in agreement with pre-clinical data that show DDR1 inhibition stimulates clearance of neurotoxic proteins via autophagy and reduces inflammation in animal models of neurodegeneration [18, 22, 25, 26, 30]. Notably, critical markers of apoptosis such as caspase-3 and BCL2 expression are reduced with nilotinib, consistent with volumetric magnetic resonance imaging (MRI) that show reduced atrophy of hippocampal volume in nilotinib-treated AD patients [33].

DDR1 plays a direct role in angiogenesis [37] and regulates the BBB [38] via interaction with collagen [39] and epithelial cell maintenance as an ECM-sensing TK [40] that controls inflammation [41]. We observed reduction of DDR1 and COL25A1, which is also known as collagen-like Alzheimer’s amyloid plaque component precursor specifically expressed in neurons and colocalized with Aβ plaques in AD brains [42, 43]. *COL25A1* increases AD risk in a Swedish population [44]. COL25A1 is a type of collagen expressed by neurons that interacts with BACE1 that cleaves APP, and induces synapse damage and astrocyte activation [45]. The *COL4A1* gene, in association with COL4A3, are necessary for maturation of type IV collagen, and COL4A1 mutations cause cerebral small vessel disease, i.e., intracerebral hemorrhage and shortened life-span [46, 47]. COL4A1 mutations are associated with increased thickening of capillary basement membranes [47], suggesting vascular fibrosis, in agreement with the preclinical data (Additional file 1) in APP/DDR1 mice. The differential changes in direction in COL4A1 and COL4A3 expression in the nilotinib-treated group may be due to subunits being associated with distinct processes that regulate overall collagen IV levels. Interestingly, DDR1 activation is required for Collagen IV synthesis [48] and DDR1 regulates collagen transcription and MMP production [49]; we observed alterations in subtypes of tissue TIMP3 and TIMP4 as key regulators of MMP expression [50] in the nilotinib group. We also observed reduction of TGF, which is the master regulator of fibrosis [51], as well as attenuation of inflammatory markers, including chemokines, cytokines and TNF-alpha, in the nilotinib-treated group in agreement with reduced collagen and fibrosis, in association with cytokines [52] and chemokine production [53]. COL6 genes encode the ECM protein collagen VI, and mutations in these genes are associated with muscular dystrophies [54].

**Fig. 6:**
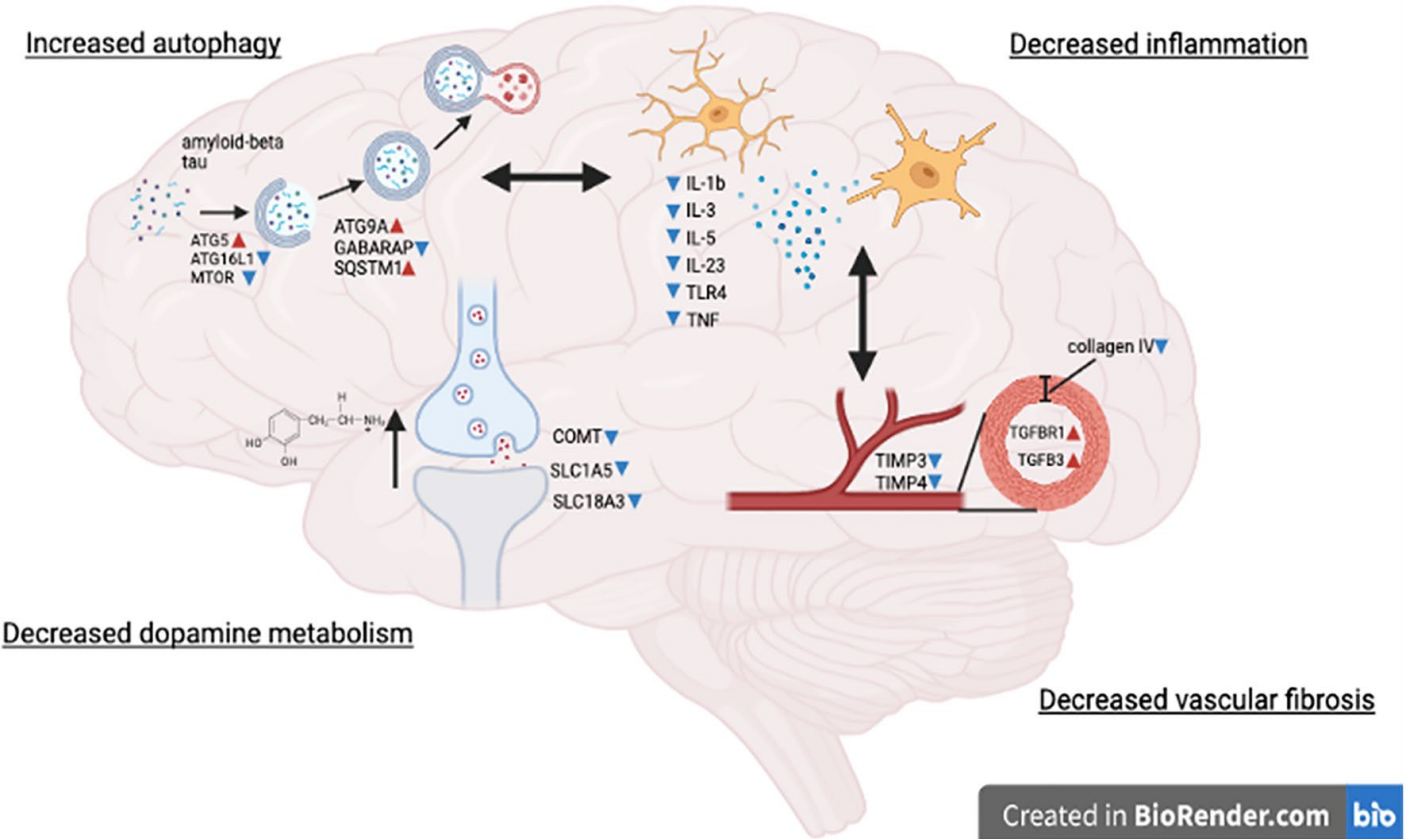
Tyrosine kinase DDR1 inhibition with nilotinib. Treatment of AD patients with nilotinib may induce multiple mechanisms that include activation of genes that control the execution and completion of autophagy, suggesting clearance of intracellular amyloid and tau that would perhaps reduce plaque burden and affect plasma amyloid and tau. Nilotinib treatment led to downregulation of several pro-inflammatory makers along with reduction of collagen, TIMPs and TGFs that maintain blood vessel integrity and prevent vascular fibrosis. Nilotinib treatment leads to reduction of dopamine breakdown probably via altered vesicular transport and reduction of COMT

Dopamine plays an important role in executive functions via dopaminergic neurons in the mesolimbic system and this role may be particularly relevant to AD [55, 56], as nilotinib prevents loss of dopamine in the ventral tegmental area (VTA) and improves cognition in AD models [57]. Indeed, AD and PD studies support a dose-dependent increase of CNS dopamine via measurement of CSF metabolites in individuals treated with nilotinib [33, 58–60], suggesting that the increases in CNS dopamine [31] with 300 mg nilotinib-treated AD patients may lead to behavioral changes [61]. Importantly, no mood swings or behavioral changes were reported in patients treated with nilotinib, 150 mg, indicating that this dosage is more adequate to avoid behavioral adverse effects in AD [33]. Our current miRNA data is consistent with previous reports showing reduction of dopamine catabolism [33] perhaps due to downregulation of COMT expression and reduced dopamine transport, which result in increased dopamine bioavailability. The miRNA results also show an effect on acetylcholine metabolism, downstream calcium signaling and early gene expression, pointing to possible nilotinib effects on neurotransmitters, including dopamine and acetylcholine. Collectively, these data indicate that nilotinib penetrates the BBB to inhibit DDR1 and alter CNS dopamine metabolism (Fig. 6).

Demonstration of miRNA changes that control genes which regulate molecular pathways involved in autophagy are indicative of autophagosome maturation, membrane trafficking and cellular transport [35] and are consistent with previous data showing the effects of nilotinib on ubiquitination and the endosomal and autophagy–lysosomal pathways [30]. Importantly, the increase in autophagy flux leads to reduction (or enhanced clearance) of intracellular or intraneuronal protein levels, including Aβ and tau, in animal models [18, 19, 62–64], suggesting that the observed reduction on Aβ PET and CSF and plasma Aβ and tau in AD patients may be due to the effect of nilotinib on neuronal autophagy (Fig. 6). Nilotinib effects on Aβ clearance provide a stark contrast with the effects of anti-amyloid therapies, e.g., Lecanemab, that targets extracellular amyloid species and only partially slows cognitive decline (27%) in early AD patients despite elimination of extracellular plaques [65]. Reduction of intracellular Aβ and tau via autophagy may also result in reduction of extracellular Aβ due to possible degradation of extracellular Aβ oligomers that internalize into endosomes/lysosomes in neurons [66] or the microglial endocytic pathway [67], suggesting that nilotinib enhances neuronal and microglial lysosome-mediated Aβ degradation.

The current miRNA analysis has several limitations due to underpowered study design that would consider more adequate inclusion and stratification of other co-morbidities of AD, such as diabetes, cerebrovascular disease, and other factors, such as sex differences and ApoE genotypes. Furthermore, evaluating extracellular vesicles in CSF following nilotinib delivery is highly desirable in future studies.

In conclusion, the tyrosine kinase inhibitor nilotinib enters the CNS, inhibits DDR1, and is associated with diverse tissue changes (Fig. 6) not only on intracellular amyloid and tau clearance but also on neuroinflammation and cerebrovascular fibrosis. Much more work is needed to prove whether diverse tissue changes are (i) reproducible, (ii) a direct or indirect effect of the drug nilotinib, and (iii) occur in significant numbers of persons. Considering that anti-amyloid antibodies, including, Lecanemab, may result in adverse effects including intracerebral hemorrhage and edema in a subset (18%) of patients [65]. Nilotinib is available on market and safe in humans, and our data support investigation of this drug as potential combination therapy with nilotinib with Lecanemab..

## Supplementary Information

**Additional file 1. **Additional methods and results.

**Additional file 2: Fig. S1.** Collagen 4and amyloid-betastaining in the cortex of 5-month-old male and female **A**–**D** APPtg/tgDDR−/−mice and **E**–**H** age matched APPtg/tg DDR+/+littermate controls, and quantification of **I** vessel wall thickness and **J** vessel diameter. **A**–**C** and **E**–**G** blood vessels were imaged longitudinally, **D** and **H** blood vessels were imaged cross-sectionally. N=4 mice per group, **=0.0048, ***=0.0002, unpaired two-tailed students *t* test.

## Data Availability

The data supporting the current study are available from the corresponding author on request.

## Abbreviations

miRNA: MicroRNA
DDR: Discoidin domain receptor
MMP: Matrix metalloproteases
BBB: Blood–brain barrier
TKI: Tyrosine kinase inhibition
IC_50_: Half-maximal inhibitor concentration
CML: Chronic myelogenous leukemia
UMI: Unique molecular identifier
DEM: Differentially expressed miRNA
GO: Gene ontology
ECM: Extracellular matrix
VEGF: Vascular endothelial growth factor
MCI: Mild cognitive impairment
