# Supplementary material for: Inhibition of discoidin domain receptor (DDR)-1 with nilotinib alters CSF miRNAs and is associated with reduced inflammation and vascular fibrosis in Alzheimer’s disease
Source: J Neuroinflammation. 2023 May 16;20:116. doi: 10.1186/s12974-023-02802-0 (PMC10186647; doi:10.1186/s12974-023-02802-0)
Supplement: Supplementary file 1 — Additional file 1. Additional methods and results. [file 12974_2023_2802_MOESM1_ESM.docx]

**Additional file 1**

**Methods**

**Generation of DDR1 knockout crossed with APP mice.**

Male and female homozygous TgAPP mouse, which express the Swedish (K670N/M671L, Dutch E693Q, and Iowa D694N mutations [1] is an excellent model of AD that expresses familial APP mutations that are associated with vascular defects in AD. Male and female homozygous DDR1 knockout (DDR1^-/-^) mice [2, 3] mice were crossed with male and female homozygous TgAPP mice, which express neuronally derived human APP gene, 770 isoform, containing the Swedish (K670N/M671L, Dutch E693Q, and Iowa D694N mutations under the control of the mouse thymus cell antigen 1, theta, Thy1, promotor [1]. Breeding of TgAPP and DDR1^-/-^ yielded first generation (F1) of APP^n/tg^ DDR^+/-^ mice, which were used for second generation (F2) of the desired DDR1 WT (APP^tg/tg^ DDR^+/+^), APP on partial DDR (APP^tg/tg^ DDR^+/-^) and complete DDR (APP^tg/tg^DDR^-/-^) deletion. APP^n/tg^ DDR^+/-^ mice (F1), were male and female breeders equally from both genotypes (DDR^-/-^ and TgAPP). An F2 generation from breeding male and female APP^n/tg^ DDR^+/-^ yielded 8 genotypes of which 3 genotypes (APP^tg/tg^ DDR^+/+^, APP^tg/tg^ DDR^+/-^ and APP^tg/tg^DDR^-/-^) were used according to Georgetown University Animal Care and Use Committee (GUACUC) approved protocol.

APP^tg/tg^DDR^-/-^ mice and age matched wild-type APP^tg/tg^DDR^+/+^ littermate controls were aged five to six months to allow proper phenotypic expression.  The brains were harvested and fixed in 4% paraformaldehyde (PFA) and cryoprotected with 30% sucrose before being coronally sectioned at 30µm on a Leica CM1950 cryostat. Cortical sections were co-labelled using an anti-rabbit polyclonal collagen 4 primary antibody (1:250, Novus, cat. #NB120-6586) and an anti-mouse amyloid-beta (6e10, 1:500, Biolegend, cat. #803001) and AlexaFluor goat anti-Rabbit 594 and anti-mouse 488 secondaries, respectively, with a DAPI counterstain to stain amyloid-beta and brain blood vessels.  Sections were imaged at 40x and 63x on a Zeiss LSM 800 confocal microscope.  Blood vessel thickness was measured using ImageJ image analysis software (Figure 4, n=6, one-way ANOVA or Student’s *t* test was used. * p< 0.05). Six images (fields) per animal and 4-8 vessels per image or field (totaling 24-48 vessels) were measured in ImageJ using the straight-line tool to measure the width of collagen 4 signal and diameter size in both longitudinal and transection cut of vessels, but we did not differentiate arteries from veins.

**Results**

**DDR1 knockout significantly reduces collagen 4 levels in TgAPP mice.**

To validate our human genomic data in a preclinical model of AD, we generated TgAPP^tg/tg^/DDR^+/+^ that how amyloid (green) and collagen 4 (red) staining around blood vessels (Suppl Fig. 1A,B, C). Collagen4 staining (Suppl Fig. 1D) was along the endothelial lining of brain vessels. DDR1 knockout mice (TgAPP^tg/tg^/DDR^-/-^) showed amyloid (green) and vascular collagen 4 (red) staining (Suppl Fig. 1E,F, G, and H). TgAPP^tg/tg^/DDR^-/-^ display significantly reduced levels of collagen 4 along the endothelial lining of brain vessels, resulting in increased blood vessel diameter as measured in both longitudinal (Suppl Fig. 1B,F) and cross sectional (Suppl Fig. 1D, H) brain sections. This was measured by quantifying both overall vessel wall thickness as well as relative unoccluded vessel diameter (open vessel diameter/total vessel diameter).

**References**

1. Davis J, Xu F, Deane R, Romanov G, Previti ML, Zeigler K, Zlokovic BV, Van Nostrand WE: **Early-onset and robust cerebral microvascular accumulation of amyloid beta-protein in transgenic mice expressing low levels of a vasculotropic Dutch/Iowa mutant form of amyloid beta-protein precursor.** *J Biol Chem* 2004, **279:**20296-20306.

2. Schminke B, Muhammad H, Bode C, Sadowski B, Gerter R, Gersdorff N, Burgers R, Monsonego-Ornan E, Rosen V, Miosge N: **A discoidin domain receptor 1 knock-out mouse as a novel model for osteoarthritis of the temporomandibular joint.** *Cell Mol Life Sci* 2014, **71:**1081-1096.

3. Vogel WF, Aszodi A, Alves F, Pawson T: **Discoidin domain receptor 1 tyrosine kinase has an essential role in mammary gland development.** *Mol Cell Biol* 2001, **21:**2906-2917.
