# Supplementary material for: Inhibition of discoidin domain receptor (DDR)-1 with nilotinib alters CSF miRNAs and is associated with reduced inflammation and vascular fibrosis in Alzheimer’s disease
Source: J Neuroinflammation. 2023 May 16;20:116. doi: 10.1186/s12974-023-02802-0 (PMC10186647; doi:10.1186/s12974-023-02802-0)
Supplement: Supplementary file 2 — Additional file 2: Fig. S1. Collagen 4and amyloid-betastaining in the cortex of 5-month-old male and female A–D APPtg/tgDDR−/−mice and E–H age matched APPtg/tg DDR+/+littermate controls, and quantification of I vessel wall thickness and J vessel diameter. A–C and E–G blood vessels were imaged longitudinally, D and H blood vessels were imaged cross-sectionally. N=4 mice per group, **=0.0048, ***=0.0002, unpaired two-tailed students t test. [file 12974_2023_2802_MOESM2_ESM.pdf]

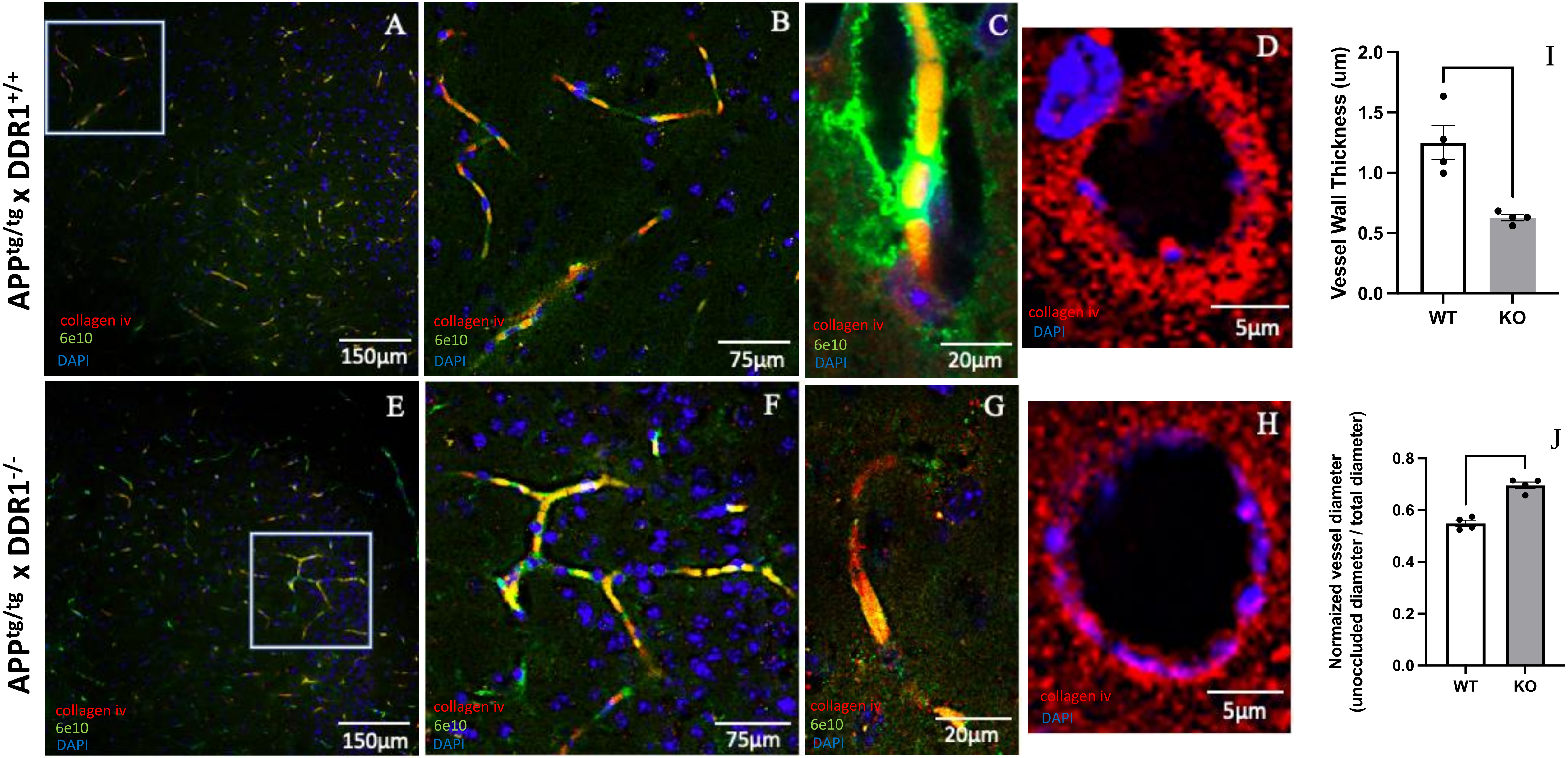

Supplemental Figure 4- Collagen 4 (red, AlexaFluor 594) and amyloid-beta (6e10, green, AlexaFluor 488) staining in the cortex of 5-month old male and female A-D) $APP^{tg/tg}DDR^{-/-}$ (KO) mice and E-H) age matched $APP^{tg/tg}$ $DDR^{+/+}$ (WT) littermate controls, and quantification of I) vessel wall thickness and J) vessel diameter. A-C and E-G blood vessels were imaged longitudinally, D and H blood vessels were imaged cross-sectionally. N=4 mice per group, **=0.0048, ***=0.0002, unpaired two-tailed students t-test
